# Comparison of geriatric nutritional risk index and creatinine index in short-term mortality prediction in maintenance hemodialysis patients

**DOI:** 10.55730/1300-0144.5356

**Published:** 2022-01-04

**Authors:** Fatma AYERDEN EBİNÇ, Gülay ULUSAL OKYAY, Serpil Müge DEĞER, Hatice ŞAHİN, Tamer SELEN, Çise KANAR DOĞAN, Kadir Gökhan ATILGAN, Ebru GÖK OĞUZ, Mehmet Deniz AYLI

**Affiliations:** 1 Division of Nephrology, Department of Internal Medicine, Dışkapı Yıldırım Beyazıt Education and Research Hospital, University of Health Sciences, Ankara, Turkey; 2 Division of Nephrology, Department of Internal Medicine, Faculty of Medicine, Dokuz Eylül University, İzmir, Turkey

**Keywords:** Geriatric nutritional risk index, creatinine index, short-term mortality, hemodialysis patients

## Abstract

**Background/aim:**

The aim of this study is to analyze and compare the predictive values of the Geriatric Nutritional Risk Index (GNRI) and Creatinine Index (CI) in the short-term mortality of maintenance hemodialysis patients and to determine their best cut-offs.

**Material and Methods:**

A total of 169 adult hemodialysis patients were included in this retrospective, cross-sectional, and single-center study. The demographic, clinical, and laboratory data of the month in which the patients were included in the study were obtained from their medical files and computer records. All-cause death was the primary outcome of the study during a 12-month follow-up after baseline GNRI and CI calculations.

**Results:**

The mean age of the study population was 57 ± 16 years (49.7% were women, 15% were diabetic). During the one-year observation period, 19 (11.24%) of the cases died (8 CV deaths). The optimal cut-off value for GNRI was determined as 104.2 by ROC analysis [AUC = 0.682 ± 0.06, (95% CI, 0.549–0.815), p = 0.01]. The low GNRI group had a higher risk for all-cause and CV mortality compared to the higher GNRI group (p = 0.02 for both in log-rank test). The optimal sex-specific cut-off was 12.18 mg/kg/day for men [AUC = 0.723 ± 0.07, (95% CI, 0.574–0.875), p = 0.03] and was 12.08 mg/kg/day for females [AUC = 0.649 ± 0.13, (95% CI, 0.384–0.914), p = 0.01]. Patients with lower sex-specific CI values had higher all-cause and CV mortality (p = 0.001 and p = 0.009 in log-rank test, respectively). In multivariate cox models, both GNRI [HR = 4.904 (% 95 CI, 1.77–13.56), p = 0.002] and sex-specific CI [HR = 5.1 (95% CI, 1.38–18.9), p = 0.01] predicted all-cause mortality. The association of GNRI with CV was lost [HR = 2.6 (CI 95%, 0.54–13.455), p = 0.22], but low CI had a very strong association with CV mortality [HR = 11.48 (CI 95%, 1.25 −104), p = 0.03].

**Conclusion:**

In hemodialysis patients, GNRI and CI have similar powers in predicting all-cause short-term mortality. The association of CI with all-cause death depends on gender. On the other hand, sex-specific CI predicts CV mortality better than GNRI.

## 1. Introduction

Malnutrition is a common and complex complication that can develop in maintenance hemodialysis (MHD) patients [1]. It can lead to life-threatening conditions such as inflammation, infections, immune, and cardiovascular (CV) dysfunctions [2]. Thus, it is exceedingly important to follow the nutritional status with effective techniques and to identify any problems in a timely manner [3, 4]. It is crucial to prevent and/or to treat malnutrition in order to reduce the complications of primary disorder and to improve either quality of life or life expectancy [3].

Geriatric Nutrition Risk Index (GNRI) and Creatinine Index (CI) are new nutritional indices calculated using different variables that have superiority over each other at some points [5]. For example, while the GNRI defines malnutrition as much stronger than hypoalbuminemia, hypocholesterolemia, and low body mass index, it is claimed that CI better reflects dietary protein intake and skeletal muscle mass [5–9]. A limited number of studies have documented the predictive power of mortality of GNRI and CI in different patient settings [10–13]. The number of studies evaluating the potential differences of these indices in the same population is even less [14]. Such a comparison may provide clearer results by reducing the effects of study conditions and/or population characteristics on the results.

GNRI and CI can be readily calculated with the parameters used in the routine follow-up of hemodialysis patients. We think that both can provide practical information for assessing the nutritional status and mortality rate of these patients. Based on these data and hypotheses, we designed this study to investigate and compare the predictive values of GNRI and CI on short-term mortality in maintenance hemodialysis patients and to determine optimal cut-off points.

## 2. Materials and methods

All adult (> 18 years old) patients who received MHD at the Dışkapı Yıldırım Beyazıt Training and Research Hospital between January 1 and December 31, 2017, were evaluated retrospectively for eligibility for the study. Those who have been on MHD for less than 6 months, those with liver disease, active malignancy, or autoimmune disease, those who had active infection or inflammation within 3 months, those with urine output above 100 cc/day, those having dialysis sessions other than 3 days a week (1, 2 or 4 days) were excluded from the analysis. Finally, 169 patients were included in the study. The study was started after the approval of the resident Medical Ethics Committee and was administered under the protocols of the Declaration of Helsinki.

Demographic, anthropometric, and laboratory data of the month in which the patients were included in the study were got from medical files and computer records. Patients were examined for mortality during the 12-month follow-up period, and, if developed, causes of death were noted. Age, sex, body weight, height, dialysis duration, presence of diabetes, spKt/Vurea (single-pool urea clearance index for weekly dialysis dose; K: urea clearance, t: dialysis time and V: urea distribution volume), hemoglobin, fasting blood glucose (FBG), urea, serum creatinine, albumin (alb), calcium (Ca), phosphorus (P), total cholesterol, low-density lipoprotein (LDL)-cholesterol, triglyceride, and C-reactive protein (CRP) values were recorded. Body mass index (BMI) was calculated by dividing weight (kg) by height (m) square.

The Geriatric Nutrition Risk Index (GNRI), defined by Boulline et al. [15] for use in elderly patients, has been revised by Yamada for use in dialysis patients [16]. In this study, GNRI was calculated by the formula of [GNRI = 1.489 X serum albumin (g / L) + (41.7 X current weight (kg) / ideal weight (kg))] using height, weight, and serum albumin level.

Modified creatinine index (CI) is a relatively new index described by Canaud et al., which has been shown to be a reliable marker for measuring lean body mass [8]. In this study, it was calculated using age, sex, predialysis serum creatinine and spKt/Vurea with the following formula of [CI (mg/kg/day) = 16.21 + (1.12 X [1, if male; 0, if female]) – (0.06 X age (years)) – (0.08 X spKt/Vurea) + (0.009 X creatinine (mol/L))].

## 3. Statistical analysis

Statistical analyzes were performed using the 24th version of the Statistical Package for the Social Sciences software (SPSS Inc., Chicago, IL, USA). The distribution of numerical variables was evaluated using the Kolmogorov–Smirnov test. The normally distributed continuous data were compared with Student’s t-test, and the results of the tests were given as mean values ± standard deviation (SD). Abnormally distributed data were compared with the Mann–Whitney U test and results were presented as the median and interquartile range (IQR). Comparisons of categorical variables were made using the Chi-square test and Fisher’s exact test; results were presented as numbers and percentages.

Receiver operating characteristic (ROC) curve analysis was applied to estimate the optimum GNRI and CI thresholds that could predict all-cause mortality. Cut-off values for CI were calculated separately for men and women. In all tests, GNRI and CI were evaluated as both continuous variables and categorical variables (according to cut-off values). Survival analyzes were performed using Kaplan–Meier survival curves, and the duration was chosen as 12 months. Univariate and multivariate cox-proportional hazard models were applied to identify factors associated with all-cause mortality and CV mortality. Logarithmically transformed values were used for data not normally distributed in Cox regression analyses. Since the number of events in the study group was limited, multivariate models were created by adjusting for age and sex. Results were presented with hazard ratio (HR) and 95% confidence intervals (CI).

## 4. Results

The main characteristics of 169 MHD patients included in the study are summarized in Table-1. The mean age of the study population was 57 ± 16 years. A total of 49.7% were women and 15% were diabetic. The median dialysis duration was 33 (IQR, 23–60) months. The mean GNRI was 105.9 ± 16.6, and the median CI was 12.6 (IQR, 11.8–13.2) mg/kg/day.

Characteristics of surviving and nonsurviving patients are presented in Table 1. During the one-year study period, 19 (11.24%) deaths were recorded. 8 of these were due to CV events. Survivors had lower hemoglobin (p = 0.03), serum albumin (p = 0.006), total cholesterol (p = 0.014), LDL-cholesterol (p = 0.018), GNRI (p = 0.01), CI (p = 0.03), and higher CRP (p = 0.02).

The optimal cut-off for GNRI was determined by ROC analysis (64% sensitivity, 60% specificity) as 104.2 [AUC = 0.682 ± 0.06, (95% CI, 0.549 to 0.815), p = 0.01]. When the patients were divided into two groups according to this cut-off value, 70 were in the low GNRI group and 99 were in the high GNRI group. During the 12-month follow-up period, 13 patients in the low GNRI group and 6 patients in the high GNRI group died (Table-1, p = 0.02 in the chi-square test). 5 patients in the low GNRI group and 3 patients in the high GNRI group died from CV causes (Table-1, p = 0.29 in the chi-square test). Kaplan Meier survival curves showed that patients in the low GNRI group had a higher risk of all-cause mortality (Figures-a, b,c,d) and CV mortality (Figures-a,b,c,d) (p < 0.05 in the log-rank test, for both).

The optimal cut-off values for CI were calculated separately for males and females and called sex-specific CI cut-offs. Optimal sex-specific CI cut-off was 12.18 mg/kg/day for males (75% sensitivity, 65% specificity) [AUC = 0.723 ± 0.07, (95% CI, 0.574 to 0.875), p = 0.03] and, 12.08 mg/kg/day for females (75% sensitivity, 60% specificity) [AUC = 0.649 ± 0.13, (95% CI, 0.384 to 0.914), p = 0.01]. 69 patients were in the low and 100 patients in the high CI group. There were 15 deaths in the low CI group and 4 deaths in the high CI group (Table-1, p = 0.002 in the chi-square test). 7 of the deaths in the low group and 1 of the deaths in the high CI group were because of CV causes (Table-1, p = 0.02 in the chi-square test). Kaplan Meier survival curve analyses showed that patients with low sex-specific CI values had higher all-cause (Figures-a,b,c,d) and CV mortality (Figures-a,b,c,d) compared to the high sex-specific CI values (p < 0.05 in the log-rank test, for both).

### 4.1. The predictors of all-cause mortality

In univariate cox regression analyses, hemoglobin, albumin, total cholesterol, LDL-cholesterol, CRP, GNRI, and CI were associated with all-cause mortality. Specifically, higher GNRI [HR = 0.93, (CI 95%, 0.90–0.97), p < 0.001] and higher CI values [HR = 0.01, (CI 95%, 0.01–0.75), p = 0.04] were protective (Table-2). In the second analyzes (with GNRI and CI as categorical variables based on cut-off values determined by ROC analysis), lower GNRI [HR = 5.195 (95% CI, 1.92–13.99), p = 0.001] and lower sex-specific CI [ HR = 5.01, (CI 95%, 1.15–15.0), p = 0.004] were also strongly associated with all-cause mortality.

In age- and sex-adjusted multivariate cox models, all-cause mortality was associated with hemoglobin, CRP, albumin, and GNRI [HR=0.93, (CI 95% (0.90–0.97) p < 0.001), but not with CI [HR = 1.09, (CI 95%, 0.00–2.85), p = 0.36] (Table-2). In second analyzes using GNRI and CI as categorical variables, both GNRI [HR = 4.904, (CI 95, 1.77–13.56), p = 0.002] and sex-specific CI [HR = 5.1, (CI 95%, 1.38–18.9), p = 0.01] were associated with all-cause mortality.

### 4.2. Predictors of cardiovascular mortality

In univariate cox regression models, CV-mortality was associated with age, diabetes mellitus, serum creatinine, albumin, total cholesterol, LDL-cholesterol, triglycerides, CRP, GNRI, and CI. Higher GNRI [HR = 0.93, (CI 95%, 0.89–0.98), p = 0.01], and CI levels [HR = 0.20, (CI 95%, 0.07–0.56), p = 0.002] were protective against CV mortality. In analyzes using categorical variables, both low GNRI [HR = 4.71, (CI 95%, 1.067–20.83), p = 0.04] and low sex-specific CI [HR = 11.4 (CI 95%, 1.25–104), p = 0.03] were identified as predictors of CV mortality.

The association of diabetes mellitus, serum creatinine, albumin, CRP, GNRI, and CI levels with CV mortality continued in age and sex-adjusted multivariate models (Table-2). High GNRI levels [HR = 0.93 (CI 95%, 0.88–0.99), p = 0.03] and high CI levels [HR = 0.18 (CI 95%, 0.06–0.54), p = 0.002] were found to be protective against CV mortality. However, in the second analyzes using categorical variables, the association between GNRI and CV mortality disappeared [HR = 2.6 (CI 95%, 0.54–13,455), p = 0.22]. However, low sex-specific CI was found to be very strongly associated with CV mortality [HR = 11.48 (CI 95%, 1.25–104), p = 0.03].

## 5. Discussion

In this study, we investigated the association between two nutritional markers and mortality in MHD cases. We showed that both GNRI and CI were predictors for all-cause short-term mortality. We determined the optimal cut-off for GNRI as 104.2 and sex-specific CI as 12.08 mg/kg/day and 12.83 mg/kg/day for women and men, respectively. According to our analyses, sex-specific CI predicted CV mortality better than GNRI. Besides these markers, serum albumin and CRP were also associated with mortality in these patients, parallel to the previous literature.

In this study, we determined that GNRI predicted all-cause short-term mortality in MHD patients. This result has been previously reported in the literature [6,10,12]. However, there was no set threshold value for this index. We determined the optimum GNRI threshold for the risk of death from all causes in our cohort to be 104.2. GNRI values below this value were associated with shorter survival times and a 5-fold increased risk of death. However, the association between GNRI and CV mortality was not that strong. High GNRI values (continuously variable) were only 7% protective against CV mortality in regression analysis, and this weak association lost statistical significance in categorical analysis. We thought that the GNRI threshold we determined for all-cause mortality might not predict CV mortality. In addition, we considered that only 8 CV-deaths might have prevented the emergence of a potential relationship. However, in literature reviews, strong associations between GNRI-all-cause mortality were remarkable in the European and Japanese dialysis cohorts, without a GNRI-CV mortality relationship [17,18]. Other factors such as inflammation and oxidative stress may also influence CV mortality [19,20]. Further studies in larger cohorts will help to explore the details of the link between GNRI and CV mortality.

Some researchers suggest that monitoring changes in muscle mass may be a practical approach to managing the risk of death in dialysis patients [21,22]. Some other researchers assert CI can yield meaningful information about functional muscle mass in HD patients [8,9]. Based on these data, we expected to find an association between CI and all-cause mortality in our cohort. However, we failed to show such a relationship in our initial analysis. When categorical data were created by defining CI thresholds separately for men and women, shorter average life expectancy and 5 times higher risk of all-cause mortality were determined in cases with low CI values. These data were in line with previous data showing that CI may be influenced by sex [5,9,23]. Our study revealed a very strong association between CI and CV mortality. While high CI values were 80% protective against CV mortality, low CI values increased the risk 11 times. This power of CI in determining CV mortality risk maybe because it is a marker of functional muscle mass. It can be speculated that the amount of myokine synthesized from skeletal muscle may play a role in this relationship. As it is known, myokines are a group of cytokines that have receptors in many organs and have protective effects on the CV system. A change in skeletal muscle mass that causes a decrease in myokine concentration can lead to CV events and related deaths [24,25]. Our data are not designed to explain the underlying mechanisms. However, our results may shed light on further studies.

We have some important limitations. First, the retrospective design and the small number of patients would limit the conclusion of the cause-effect relationship. However, the comparison of these two indices in short-term prognosis among HD patients is an important point, since the literature is limited.

In conclusion, GNRI and CI have similar powers in predicting all-cause mortality in HD patients. When each marker is below the threshold, the risk of death in HD patients increases almost 5 times. The relation between CI and all-cause mortality depends on gender. However, the predictive power of both indices is different for CV mortality. Higher GNRI values provide 7% protection, while higher CI values provide 80% protection. Our study would provide a point of view for new prospective studies involving more HD patients.

## Figures and Tables

**Figures a, b, c, d:**
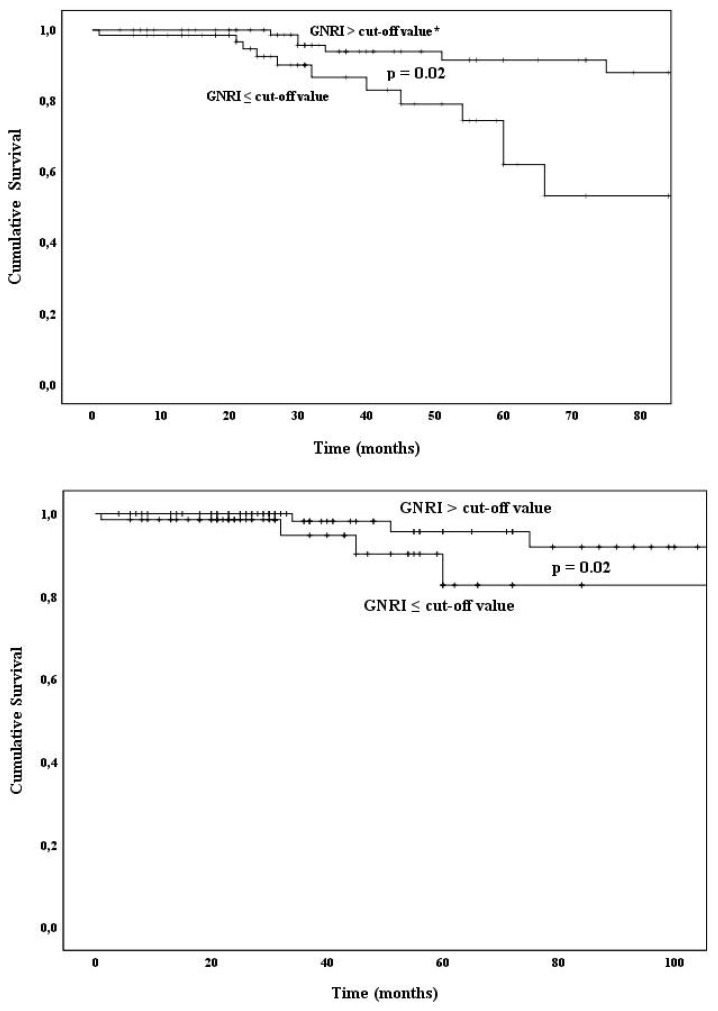

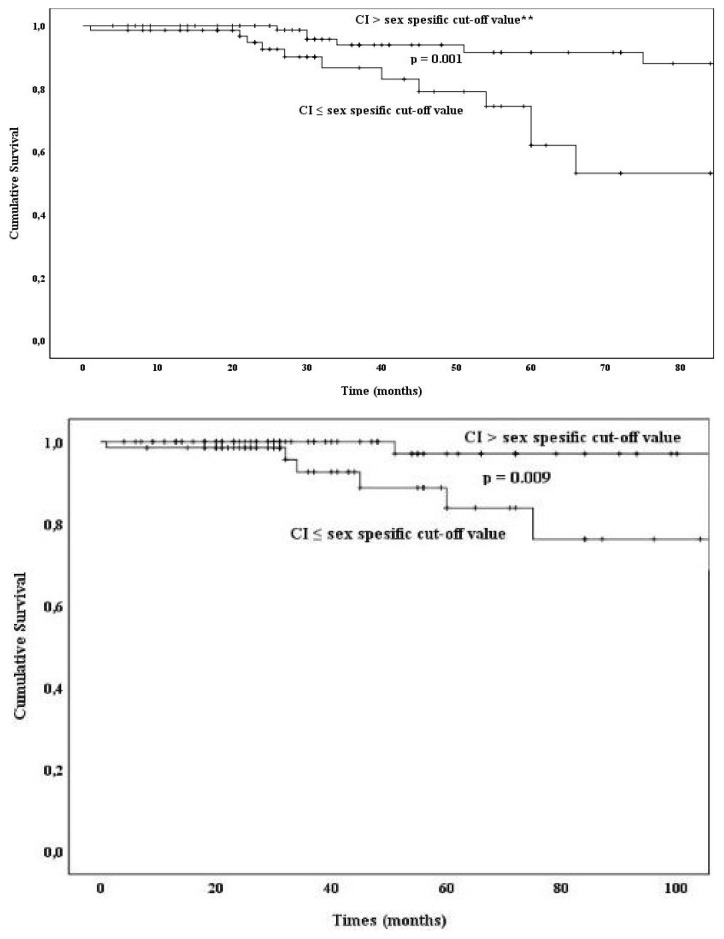
Kaplan–Meier survival curves for mortality analyses. **Figure a:** GNRI and all-cause mortality. **Figure b:** GNRI and CV mortality, **Figure c:** CI and all-cause mortality, **Figure d:** CI and CV mortality. The log-rank test was used in the analyses. Two-tailed p < 0.05 was considered statistically significant. Abbreviations: GNRI; Geriatric Nutritional Risk Index, CI; Creatinine Index, CV; cardiovascular. * GNRI cut-off determined by ROC analysis (64% sensitivity, 60% specificity) to be 104.2. ** The optimal sex-specific CI cut-off determined by ROC analysis was 12.18 mg/kg/day for males (75% sensitivity, 65% specificity) and 12.08 mg/kg/day for females (75% sensitivity, 60% specificity).

**Table-1:** Baseline characteristics of the study population and univariate comparisons of survivors and nonsurvivors.

	Study population (n = 169)	Survivors (n = 150)	nonsurvivors (n = 19)	p
Age (years)	57.09 ± 16.19	56.60 ± 15.43	61.00 ± 21.45	0.266
Sex (Male)	85 (50.3 %)	74 (49.33 %)	11 (57.89 %)	0.627
BMI (kg/m^2^)	24.6 ± 5.4	24.86 ± 5.59	23.13 ± 3.91	0.115
Diabetes Mellitus (n, %)	26 (15 %)	22 (14.66 %)	4 (21.05 %)	0.499
Hemodialysis Duration (months)	33 (23–60)	32 (23–63)	34 (26–60)	0.890
spKt/V	1.5 (1.2–1.8)	1.5 (1.2 – 1.8)	1.5 (1.15 – 1.7)	0.575
***Blood tests***				
Hemoglobin (g/dL)	10.5 ± 2.3	10.70 ± 2.4	9.48 ± 1.7	0.03
Fasting blood glucose (mg/dL)	98 (81–138)	98 (81–137)	98 (80–138)	0.79
Urea (mg/dL)	130 ± 40	131 ± 38	129 ± 49	0.85
Creatinine (mg/dL)	7.09 ± 2.4	7.14 ± 2.42	6.67 ± 2.33	0.41
Albumin (g/dL)	3.8 ± 0.6	3.8 ± 0.61	3.35 ± 0.69	0.006
Calcium(mg/dl)	8.6 ± 0.9	8.59 ± 0.96	8.59 ± 1.04	0.986
Phosphorus (mg/dL)	5.5 ± 1.6	5.56 ± 1.63	5.71 ± 2.01	0.716
Total Cholesterol (mg/dL)	175 ± 51	178 ± 51	143 ± 42	0.014
LDL-Cholesterol (mg/dL)	125 ± 42	127 ± 42	100 ± 35	0.018
Triglyceride (mg/dL)	179 ± 98	184 ± 98	141 ± 96	0.150
CRP (mg/dL)	8.5 (5.1–21)	8.2 (5–18)	18.7 (6.4–82)	0.02
Ferritin (mcg/L)	626 (377–718)	585 (362–712)	768 (486–1347)	0.304
Parathormone (pg/ml)	450 (213–630)	450 (213–635)	374 (213–635)	0.479
***Nutritional Indices***				
Geriatric Nutritional Risk Index				
Continuous variable	105.9 ± 16.6	107.07 ± 16.53	96.38 ± 15.08	0.01
Categoricalvariable (low vs. high)	70 (41.42 %) vs. 99 (58.57 %)	57 (38.0 %) vs. 93 (56.7 %)	13 (68.4 %) vs. 6 (31.6 %)	0.02
Creatinine Index (mg/kg/day)				
Continuous variable	12.6 (11.8–13.2)	12.71 (11.86–13.30)	12.03 (11.4–12.9)	0.03
Categoricalvariable (low vs. high)	69 (40.87 %) vs. 100 (59.17 %)	58 (38.66 %) vs. 92 (66.33 %)	15 (78.94 %) vs. 4 (21.05 %)	0.008

Continuous data are presented as mean ± standard deviation or median and interquartile ranges (IQR; the range of values lying between the 25th and 75th centiles) depending on their distribution. Categorical variables are shown as frequency and percentages. Abbreviations: BMI; body mass index, spKt/V; single pool urea clearance index, (K; urea clearance, t; dialysis time and V; urea distribution volume), LDL: low density, CRP: C-reactive protein.

**Table-2:** Univariate and multivariate cox regression analyses for all-cause and cardiovascular mortality.

	All-cause mortality (n = 19)						Cardiovascular mortality (n = 8)					
	Univariate			Multivariate			Univariate			Multivariate		
Variable	HR	95 % CI	p	HR	95 % CI	p	HR	95 % CI	p	HR	95 % CI	p
Age (year)	1.01	0.98, 1.04	0.26	-	-	-	1.10	1.03,1.17	0.002	-	-	-
Gender	0.65	0.26, 1.62	0.35	-	-	-	1.18	0.29, 4.78	0.80	-	-	-
BMI (kg/m^2^)	0.93	0.84, 1.04	0.25	-	-	-	0.88	0.73, 1.06	0.19	-	-	-
Diabetes Mellitus	1.88	0.62, 5.7	0.26	-	-	-	4.3	1.03, 18.3	**0.04**	8.30	1.40, 48.3	**0.02**
Hemoglobin (g/dL)	0.63	0.48, 0.83	**0.001**	0.59	0.45, 0.78	**< 0.001**	0.77	0.52,1.14	0.19	-	-	-
Albumin (g/dL)	0.17	0.09, 0.34	**< 0.001**	0.18	0.08, 0.38	**< 0.001**	0.16	0.05,0.46	**0.001**	0.19	0.05, 0.72	**0.01**
Phosphorus (mg/dL)	0.97	0.74, 1.29	0.88	-	-	-	0.83	0.53,1.29	0.41	-	-	-
Total Cholesterol (mg/dL)	0.98	0.97, 1.00	**0.04**	0.98	0.96, 1.002	0.08	0.97	0.95, 1.00	**0.04**	0.96	0.93, 1.00	0.06
LDL Cholesterol (mg/dL)	0.98	0.96, 0.99	**0.03**	0.98	0.97, 1.001	0.07	0.97	0.94, 0.99	**0.02**	0.96	0.93, 1.00	0.08
Triglyceride (mg/dL)	0.99	0.98, 1.002	0.12	-	-	-		0.96, 0.99	**0.03**	0.97	0.95, 1.00	0.11
CRP (mg/dL) *	1.02	1.01, 1.04	**< 0.001**	1.02	1.01, 1.04	**0.001**	1.02	1.00,1.04	**0.02**	1.03	1.00, 1.07	**0.03**
Ferritin(μg/L) *	1.001	0.99, 1.004	0.55	-	-	-	1.00	0.99, 1.004	-	-	-	-
Geriatric Nutritional Risk Index												
Continuous model	0.93	0.90, 0.97	**< 0.001**	0.93	0.90, 0.97	**< 0.001**	0.93	0.89,0.98	**0.01**	0.93	0.88, 0.99	**0.03**
Categorical model	5.19	1.92, 13.99	**0.001**	4.90	1.77, 13.56	**0.002**	4.71	1.06,20.83	**0.03**	2.6	0.54, 13.45	**0.22**
Creatinine Index (mg/kg/day)												
Continuous model *	0.01	0.01, 0.75	**0.04**	1.09	0.00, 2.85	0.36	0.20	0.07, 0.56	**0.002**	0.18	0.06, 0.54	**0.002**
Categorical model	5.01	1.65, 15.10	**0.004**	5.1	1.38, 18.9	**0.01**	9.67	1.18,78.77	**0.03**	11.4	1.25, 104	**0.03**

**Abbreviations:** HR; Hazard ratio, CI; Confidence interval, BMI; body mass index, LDL: low density, CRP: C-reactive protein.

* The natural logarithmic transformed values were used in analyses.

Variables with a p-value of < 0.05 in univariate analyzes were considered as independent predictor candidates for multivariate models. Multivariate models were adjusted for age and sex (since the total death event was 19, the number of variables in each model was limited to 3).
